# Comparison of a chymase inhibitor and hyaluronic acid/carboxymethylcellulose (Seprafilm) in a novel peritoneal adhesion model in rats

**DOI:** 10.1371/journal.pone.0211391

**Published:** 2019-01-25

**Authors:** Maiko Ozeki, Denan Jin, Yuta Miyaoka, Shinsuke Masubuchi, Fumitoshi Hirokawa, Michihiro Hayashi, Shinji Takai, Kazuhisa Uchiyama

**Affiliations:** 1 Department of General and Gastroenterological Surgery, Osaka Medical College, Takatsuki, Japan; 2 Department of Innovative Medicine, Graduate School of Medicine, Osaka Medical College, Takatsuki, Japan; Ehime University Graduate School of Medicine, JAPAN

## Abstract

Adhesion formation that occurred after alkali-induced injury of the cecum was used as a novel adhesion model in rats, and it was compared with that of a common adhesion model after abrading the cecum. Using the novel adhesion model, inhibition of adhesion formation by a chymase inhibitor, Suc-Val-Pro-Phe^P^(OPh)_2_, and by sodium hyaluronate/carboxymethylcellulose (Seprafilm) was evaluated, and their mechanisms were assessed. The degree of adhesion formation was more severe and more stable in the alkali-induced injury model than in the abrasion-induced injury model. Both the chymase inhibitor and Seprafilm showed significant attenuation of the degree of adhesion 14 days after alkali-induced injury. Chymase activity in the cecum was significantly increased after alkali-induced injury, but it was significantly attenuated by the chymase inhibitor and Seprafilm. Myeloperoxidase and transforming-growth factor (TGF)-β levels were significantly increased after alkali-induced injury, but they were attenuated by both the chymase inhibitor and Seprafilm. At the level of the adhesions, the numbers of both chymase-positive cells and TGF-β-positive cells were significantly increased, but their numbers were reduced by the chymase inhibitor and Seprafilm. In conclusion, a chymase inhibitor attenuated the degree of adhesions to the same degree as Seprafilm in a novel peritoneal adhesion model that was more severe and more stable than the common adhesion model, and not only the chymase inhibitor, but also Seprafilm reduced the chymase increase at the adhesions.

## Introduction

Postoperative abdominal adhesions lead to significant problems that cause bowel obstruction, abdominal pain, infertility, and difficult re-operations [1,2]. The complications affect not only quality of life but also shorten life expectancy [1]. There is general agreement that the majority of adhesions result from surgical trauma of intraperitoneal organs, but the cellular and molecular mechanisms of postoperative adhesion formation are not well understood.

Sodium hyaluronate/carboxymethylcellulose (Seprafilm) is a bioreabsorbable membrane that has been approved for prevention of adhesion formation in clinical and experimental settings [3–5]. Seprafilm is applied to the surface of tissues as a physical barrier to separate adjacent tissues, but it has no pharmacological effect [6]. On the other hand, the surgical approach, open or laparoscopic surgery, affects adhesion formation, and laparoscopic surgery may have a lower incidence of postoperative complications such as ileus than open surgery [7,8]. Recently, the use of laparoscopic surgery has increased widely because of its lower invasiveness, in addition to a lower incidence of postoperative complications. However, Seprafilm is a solid barrier film and difficult to apply through a port during laparoscopic surgery, and other barrier materials, such as a soft gel or a solution, may be needed for greater ease of use at laparoscopic surgery.

Mast cell numbers are increased around wounds in the late stage in the process of adhesion formation [9]. In mast cell-deficient mice, adhesion formation was obviously attenuated after surgery compared with normal mice [10]. Furthermore, a mast cell stabilizer also attenuated adhesion formation in rats, suggesting that mast cell-derived factors may be associated with adhesion formation [11]. Chymase is a chymotrypsin-like serine protease contained in the secretory granules of mast cells. One well-known enzymatic function of chymase is to activate transforming growth factor (TGF)-β, which is associated with adhesion formation [12]. We previously demonstrated that adhesion formation was significantly prevented by treatment with a chymase inhibitor solution just after abdominal surgery in hamsters [13]. The mechanism of chymase inhibitors is not completely clear, though it depends on its pharmacological effect.

In a general adhesion model in rats, the serosa of the cecum is abraded until punctate hemorrhage occurs, and the macroscopic adhesion score is assessed as an indicator of the degree of adhesions. However, the results of the abrasion-induced adhesion model may differ greatly among researchers. Therefore, a novel adhesion model that could show stable adhesion scores and high objectivity was developed. Next, the effects of a chymase inhibitor and Seprafilm to prevent adhesion formation were compared in this novel model, and the mechanisms were examined.

## Materials and methods

### Agents

A chymase inhibitor, Suc-Val-Pro-Phe^p^(OPh)_2_, was donated by Prof. Oleksyszyn of Wroclaw University of Science and Technology [14]. Seprafilm was purchased from Kaken Pharmaceutical Co., Ltd (Tokyo, Japan).

### Animals

All animal procedures were approved by the Committee of Animal Use and Care of Osaka Medical College (No. 30011) and performed in accordance with the Guidelines for Animal Research. Eight-week-old male Sprague–Dawley (SD) rats (n = 96) were purchased from Japan SLC (Shizuoka, Japan). Rats were housed three per cage with food and water available ad libitum, and were kept on a daily 12-hour light-dark cycle. Temperature and humidity were controlled.

### Surgical technique

**Fig 1:**
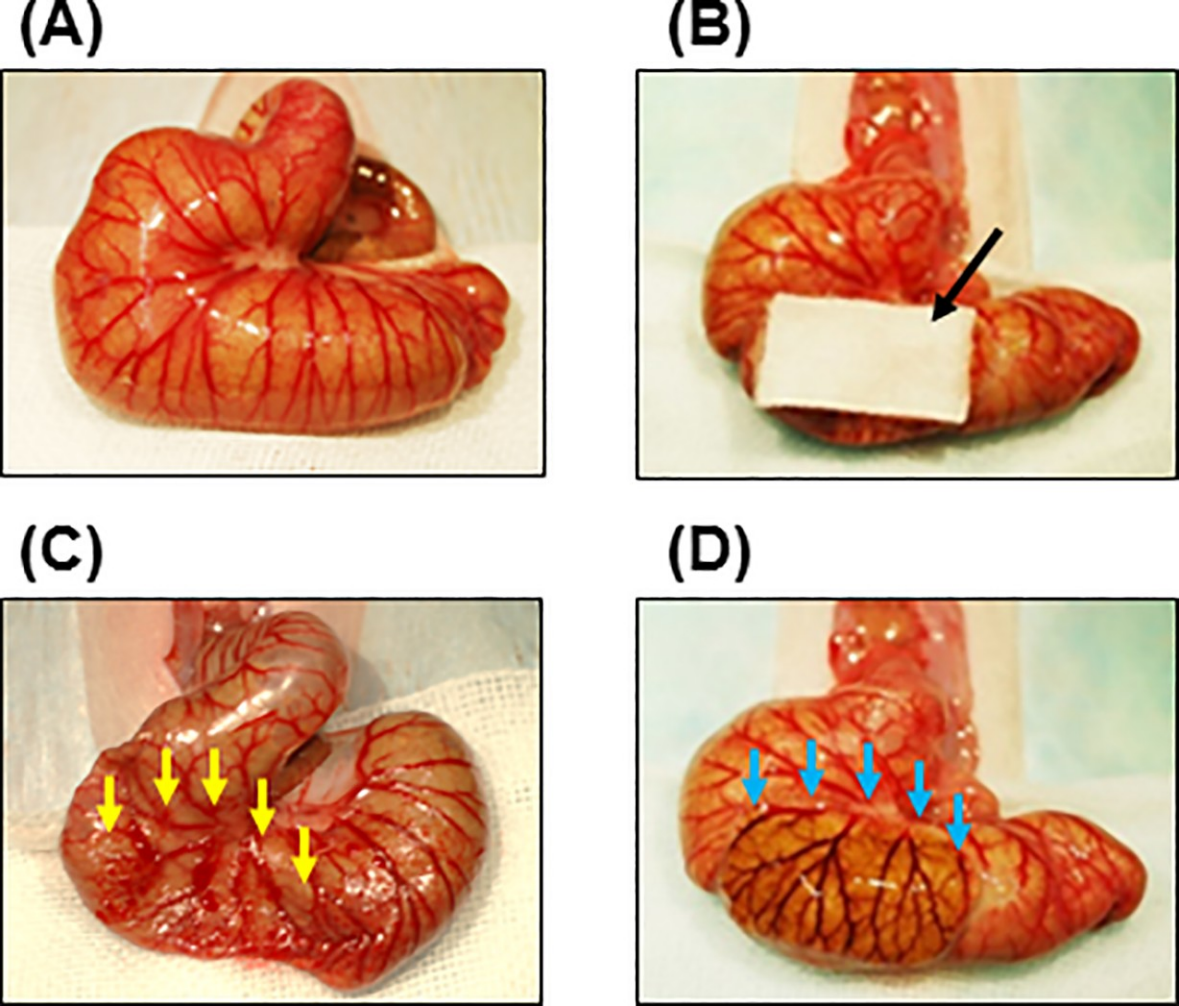
Protocol of adhesion models. Representative images of cecum in normal (A) and an alkaline (1 N sodium hydroxide)-soaked cotton pad (10 mm × 20 mm) on cecum (B). Representative images of cecums just after abrasion-injury (C) and alkali-injury (D). A black arrow shows alkaline (1 N sodium hydroxide)-soaked cotton pad (B). Yellow and blue arrows show injured regions just after abrasion with a swab (C) and alkali treatment (D), respectively.

First, the novel adhesion model was compared with a common adhesion model. Under anesthesia with inhalation of 2.5% isoflurane and 6 mg/kg xylazine hydrochloride, an abdominal midline incision was made, and the cecum was delivered on a watery gauze to keep moist in all procedures. A common adhesion model was produced according to previous papers [15,16]. The serous membrane of the cecum was abraded with a swab at area of 10 mm × 20 mm until punctate hemorrhage occurred (Fig 1). A novel adhesion model was also created by alkali-induced injury. The cecum was injured by application of an alkaline (1 N sodium hydroxide)-soaked cotton pad (10 mm × 20 mm) for 1 minute (Fig 1). The cecum was then washed out completely with saline water (Fig 1). In both models, the abdominal wall was closed in two layers with silk after the injury (Fig 1). The adhesion scores of severity and area were evaluated 1 week and 2 weeks after the injury in both models (each group, n = 6).

Next, the effects of a chymase inhibitor and Seprafilm were evaluated in the alkali-induced adhesion model. In the vehicle- or chymase-treated group, 0.2 ml of saline or 10 μM Suc-Val-Pro-Phe^p^(OPh)_2_ in saline were injected into the abdomen just after alkali-induced injury. In the Seprafilm-treated group, the alkali-injured cecum was covered with Seprafilm (10 mm × 20 mm). In the sham-operated group, a saline soaked cotton pad (10 mm × 20 mm) was put on the cecum for 1 minute, and 0.2 ml of saline were injected into the abdomen. Biochemical analysis was performed 1 day and 3 days after the injury, and the adhesion scores of adhesion and area, and histological analysis were assessed at 2 weeks (each day and each group, n = 6).

After surgical procedure, the animals recovered in a cage with free access to water and food and were monitored every 12 hours. We used power analysis, with a power of 80%, to determine the sample sizes required in each experimental group using values that have been determined in a preliminary experiment using minimum number rats.

### Scoring of adhesions

The adhesion severity scores were scored blindly according to a modified classification of Hulka et al: 0, no adhesions; 1, mild adhesions; 2, localized moderate adhesions; 3, moderate and wide adhesions; and 4, severe adhesions, impossible to separate [17]. The percent of adhesion area was calculated by the ratio of the adhered area to the initial injured area (10 mm x 20 mm), and the adhesion area scores were scored according to a modified classification of Lin et al: 0%, no adhesion; 1, 1–24% of the initial injured area; 2, 25–49% of the initial injured area; 3, 50–74% of the initial injured area; and 4, 75–100% of the initial injured area [18].

### Measurement of chymase activity

A tissue preparation method for the measurement of chymase activity has been described previously [19]. The cecum was minced and homogenized in 10 volumes (wt/vol) of 20 mmol/L Na-phosphate buffer (pH 7.4). The homogenate was then centrifuged at 8,000 rpm for 15 minutes, and supernatant was discarded. The pellets were then re-suspended and homogenized in 5 volumes (wt/vol) of 10 mmol/L Na-phosphate buffer (pH7.4) containing 2 mol/L KCL and 0.1% triton X-100. The homogenate was stored overnight at 4°C and then centrifuged at 8,000 rpm for 15 minutes, and the supernatant was used to measure chymase activity. Chymase activity in the portion of the cecum with adhesions was measured by incubating the tissue extracts with 5 mM Suc-Ala-Ala-Pro-Phe-4-Methylcoumaryl-7-amide (Peptide Institute Inc., Osaka, Japan) as the substrate. One unit of chymase activity was defined as the amount of enzyme required to cleave 1 μmol of 7-amino-4-methyl-coumarin/minute.

The protein concentration was assayed using BCA Protein Assay Reagents (Pierce, Rockford, IL, USA), with bovine serum albumin as the standard.

### Measurement of myeloperoxidase (MPO) gene expression

To measure cecal MPO gene expression, the cecum was obtained 1 day after alkali-induced injury. Total cecal RNA was extracted using the Trizol reagent (Life Technologies, Rockville, MD, USA) and subsequently dissolved in RNase-free water (Takara Bio Inc., Otsu, Japan) [20]. Total RNA (1 μg) was transcribed into cDNA with Superscript VIRO (Invitrogen, Carlsbad, CA, USA). Levels of mRNA were measured by real-time polymerase chain reaction (RT-PCR) on a Stratagene Mx3000P (Agilent Technologies, San Francisco, CA) using TaqMan fluorogenic probes. Primers and probes for RT-PCR of MPO and 18S ribosomal RNA (rRNA) were designed by Roche Diagnostics (Tokyo, Japan). The mRNA level of MPO was normalized to that of 18S rRNA.

### Measurement of TGF-β protein levels

To measure cecal transforming growth factor (TGF)-β protein levels, the cecum was collected 3 days after alkali-induced injury. The level of TGF-β was quantified using a TGF-β-specific enzyme-linked immunosorbent assay kit according to the manufacturer's instructions (R&D Systems, Minneapolis, MN).

### Histological study

A portion of the adhesions included on the uninjured side was fixed in Carnoy’s solution, embedded in paraffin, and cut into 5-μg-thick sections. The specimens were stained with hematoxylin and eosin (HE). For identification of mast cells, the specimens were stained with 0.05% toluidine blue (Chroma-Gesellschaft, Stuttgart, Germany) at pH 4.8; a red-purple-stained region was defined as mast cells [21]. To determine the localization of collagen, specimens were stained with Mallory-Azan staining; a blue-stained lesion was defined as collagen [21]. The procedure for immunohistochemical analysis of chymase has been previously described [18]. Sections were incubated with anti-chymase antibody (Cosmo Bio Co. Ltd., Hokkaido, Japan), followed by a reaction with appropriate reagents from a streptavidin-biotin peroxidase kit (Dako LSAB kit; Dako Co., Carpinteria, CA, USA) and 3-amino-9-ethylcarbazole, which was used for color development; a brown-stained region was defined as chymase-positive cells. The sections were lightly counterstained with hematoxylin.

### Statistical analysis

Data are expressed as means ± standard error of the mean (SEM) and were analyzed with BellCurve statistical analysis software for Microsoft Excel (Tokyo, Japan). Significant differences between mean values of two groups were evaluated using Student’s t-test for unpaired data. Significant differences among mean values of multiple groups were evaluated using one-way analysis of variance followed by Fisher’s protected Least Significant Difference (LSD) test. Values of P<0.05 were considered significant.

## Results

### Comparison of the novel adhesion model and a common adhesion model

The scores for adhesion severity and adhesion area in each rat are shown in Table 1.

**Table 1:** Scores for adhesion severity and adhesion area.

Rat	Adhesion severity score				Adhesion area score			
	1 week		2 weeks		1 week		2 weeks	
	Abrasion	Alkali	Abrasion	Alkali	Abrasion	Alkali	Abrasion	Alkali
1	0	4	2	4	0	4	0	4
2	0	4	0	4	0	4	0	4
3	0	4	0	4	0	4	0	4
4	1	3	0	4	1	3	0	3
5	2	2	0	3	1	3	0	4
6	0	4	3	4	0	4	1	4

**Fig 2:**
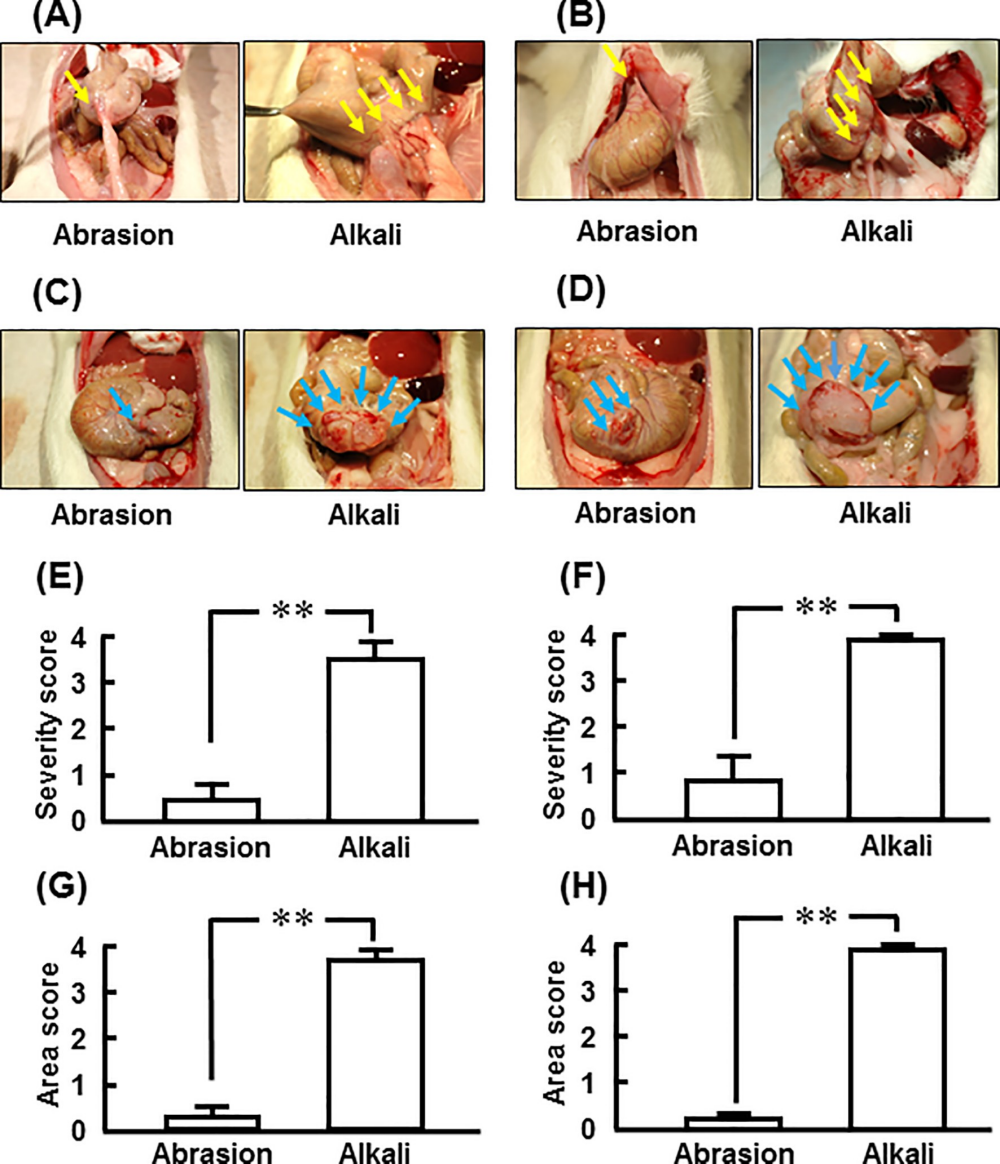
Adhesion scores for severity and area after abrasion-injury and alkali-injury. Representative cecum images 1 week (A) and 2 weeks (B) after abrasion-induced injury and alkali-induced injury. Representative cecum images when adhered tissues were removed 1 week (C) and 2 weeks (D) after abrasion-induced injury and alkali-induced injury. Adhesion severity scores 1 and 2 weeks after abrasion-induced injury (E) and alkali-induced injury (F). Adhesion area scores 1 and 2 weeks after abrasion-induced injury (G) and alkali-induced injury (H). Yellow and blue arrows show adhesion regions of the cecum (A and B) and the cecum with adhered tissues removed (C and D), respectively, 1 week and 2 weeks after abrasion-induced injury and alkali-induced injury. Values represent means ± SEM (n = 6). **P<0.01 vs. abrasion-induced injury.

Representative images of adhesion lesions 1 and 2 weeks after abrasion- and alkali-induced injuries are shown in Fig 2A and 2B, respectively. In the common adhesion model, adhesion formation was very weak 1 and 2 weeks after abrasion, but it was very strong at each point in the alkali-induced model (Fig 2A and 2B). Representative images of the abraded region and the alkali-injured region where the adhered tissues were removed from the cecum 1 week and 2 weeks after abrasion and alkali treatment are shown (Fig 2C and 2D). The adhesion area was obviously small in the abrasion-induced model compared with the alkali-induced model both at 1 week and 2 weeks (Fig 2C and 2D).

The adhesion severity score was significantly higher in the alkali-induced model than in the abrasion-induced model at 1 and 2 weeks (Fig 2E and 2F). The adhesion area score was also significantly higher in the alkali-induced model (Fig 2G and 2H).

### Effects of chymase inhibitor and Seprafilm on adhesion formation

**Fig 3:**
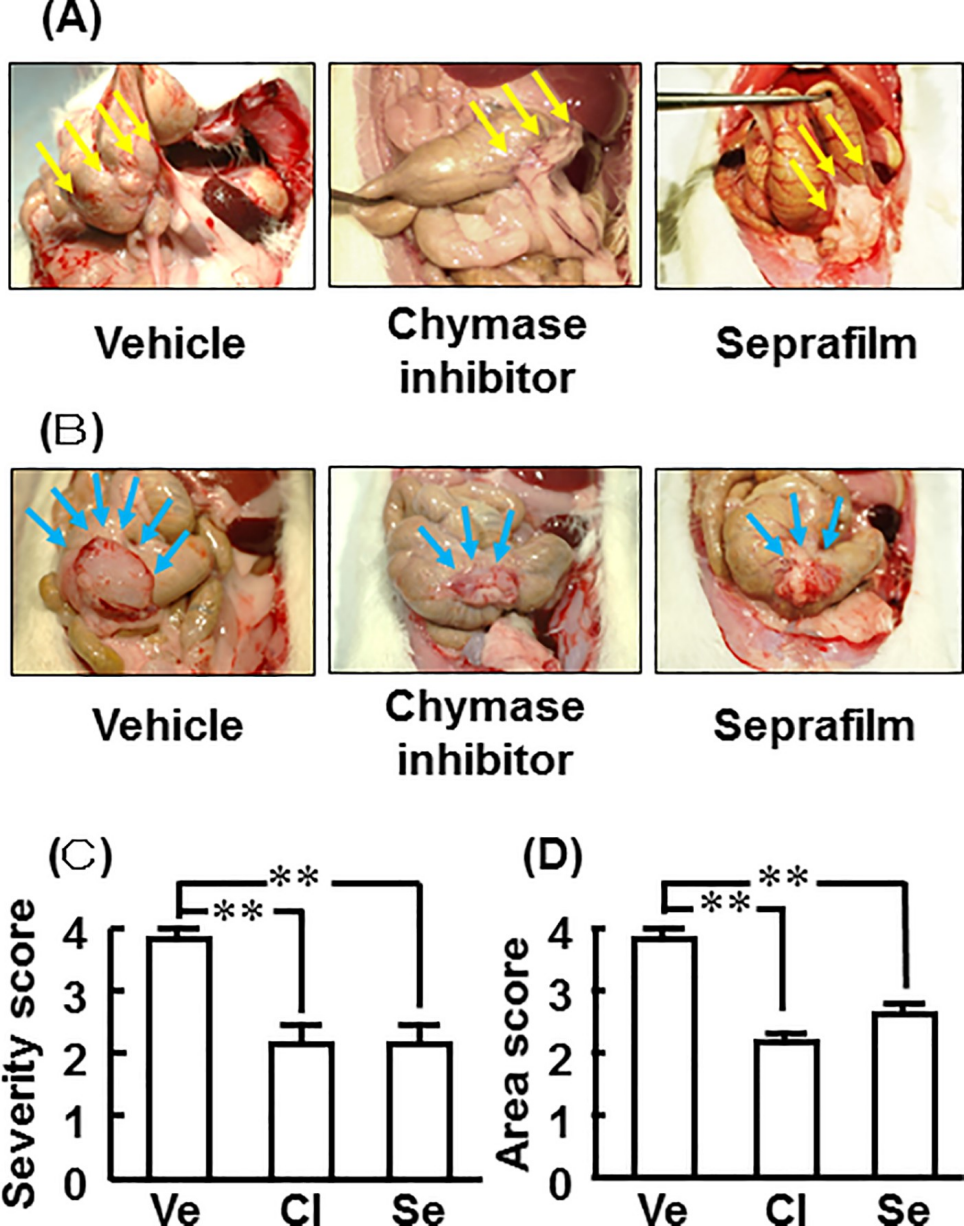
Effects of chymase inhibitor and Seprafilm on adhesion scores for severity and area. Representative cecum images from the vehicle-, chymase inhibitor-, and Seprafilm-treated rats 2 weeks after alkali-induced injury (A). Representative cecum images with adhered tissues removed from the vehicle-, chymase inhibitor-, and Seprafilm-treated rats 2 weeks after alkali-induced injury (B). Adhesion scores for severity (C) and area (D) in the vehicle (Ve)-, chymase inhibitor (CI)-, and Seprafilm (Se)-treated groups 2 weeks after alkali-injury. Yellow and blue arrows show adhesion regions of the cecum (A) and cecum with adhered tissues removed (B) 2 weeks after alkali-induced injury. Values represent means ± SEM (n = 6). **P<0.01 vs. vehicle-treated group.

Representative images of adhesion lesions 2 weeks after alkali-induced injury in the vehicle-, chymase inhibitor-, and Seprafilm-treated rats are shown in Fig 3A. Representative images of cecums with adhered tissues removed from the vehicle-, chymase inhibitor-, and Seprafilm-treated rats 2 weeks after alkali-induced injury are shown in Fig 3B.

Adhesion formation was not observed in the sham group, and adhesion severity scores were 3.83 ± 0.16, 2.1 ± 0.31, and 2.1 ± 0.31 in the vehicle, chymase inhibitor, and Seprafilm groups, respectively, 2 weeks after alkali-induced injury (Fig 3C). Adhesion area scores were 3.83 ± 0.16, 2.17 ± 0.17, and 2.67 ± 0.21 in the vehicle, chymase inhibitor, and Seprafilm groups, respectively, 2 weeks after alkali-induced injury (Fig 3D). Significant attenuation was seen in both the chymase inhibitor and Seprafilm groups compared with the vehicle group (Fig 3C and 3D).

### Cecal chymase activity

**Fig 4:**
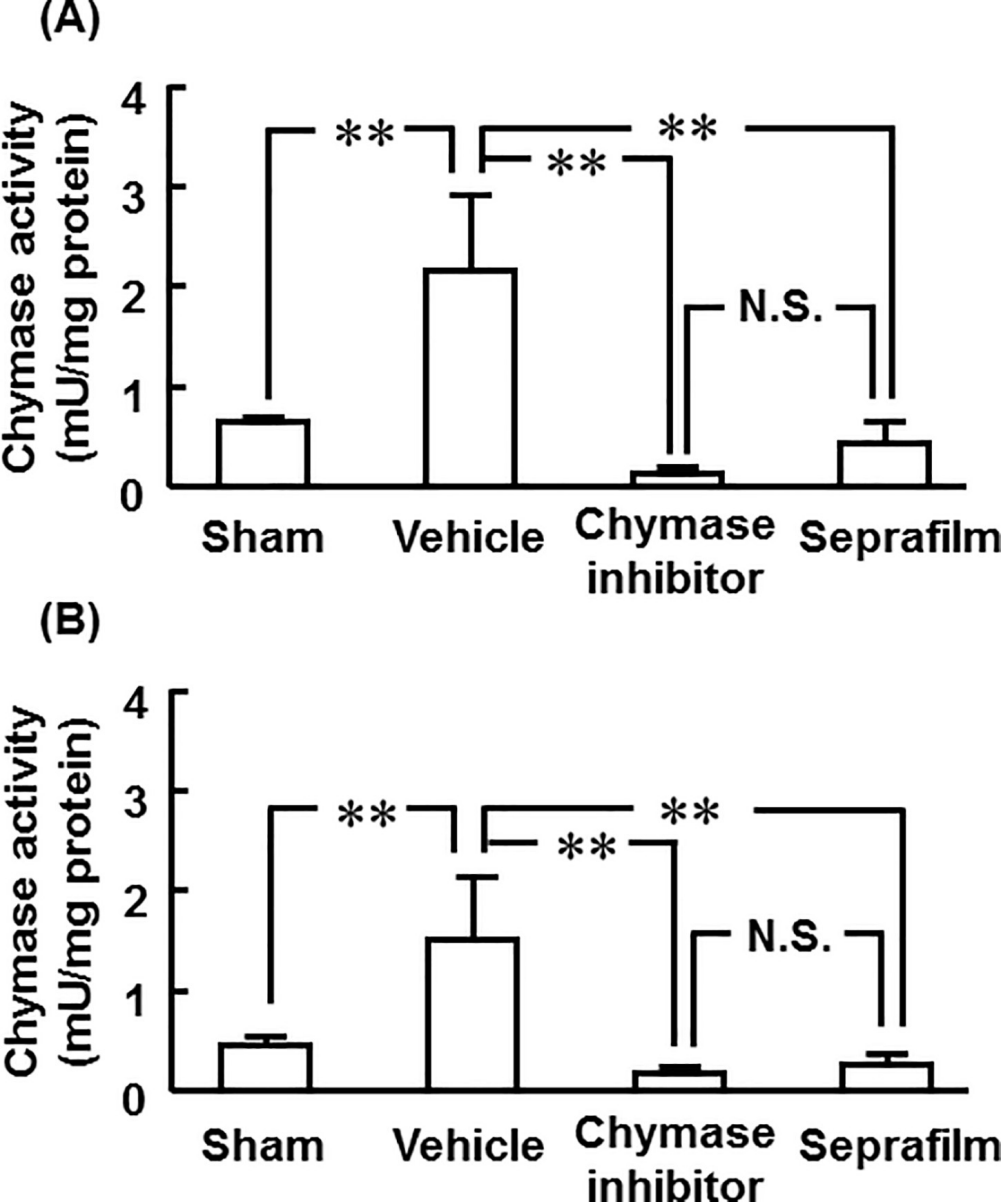
Effects of chymase inhibitor and Seprafilm on chymase activity. Cecul chymase activities in the vehicle-, chymase inhibitor-, and Seprafilm-treated groups 1 day (A) and 3 days (B) after alkali-injury. Values represent means ± SEM (n = 6). **P<0.01 vs. vehicle-treated group. N.S. means “not significant”.

Cecal chymase activity was significantly higher in the vehicle group than in the sham group 1 and 3 days after alkali-induced injury (Fig 4A and 4B). However, cecal chymase activity was significantly reduced by not only treatment with chymase inhibitor, but also by Seprafilm (Fig 4A and 4B). No significant differences between chymase inhibitor and Seprafilm were observed 1 and 3 days after alkali-induced injury (Fig 4A and 4B).

### Cecal MPO gene expression

**Fig 5:**
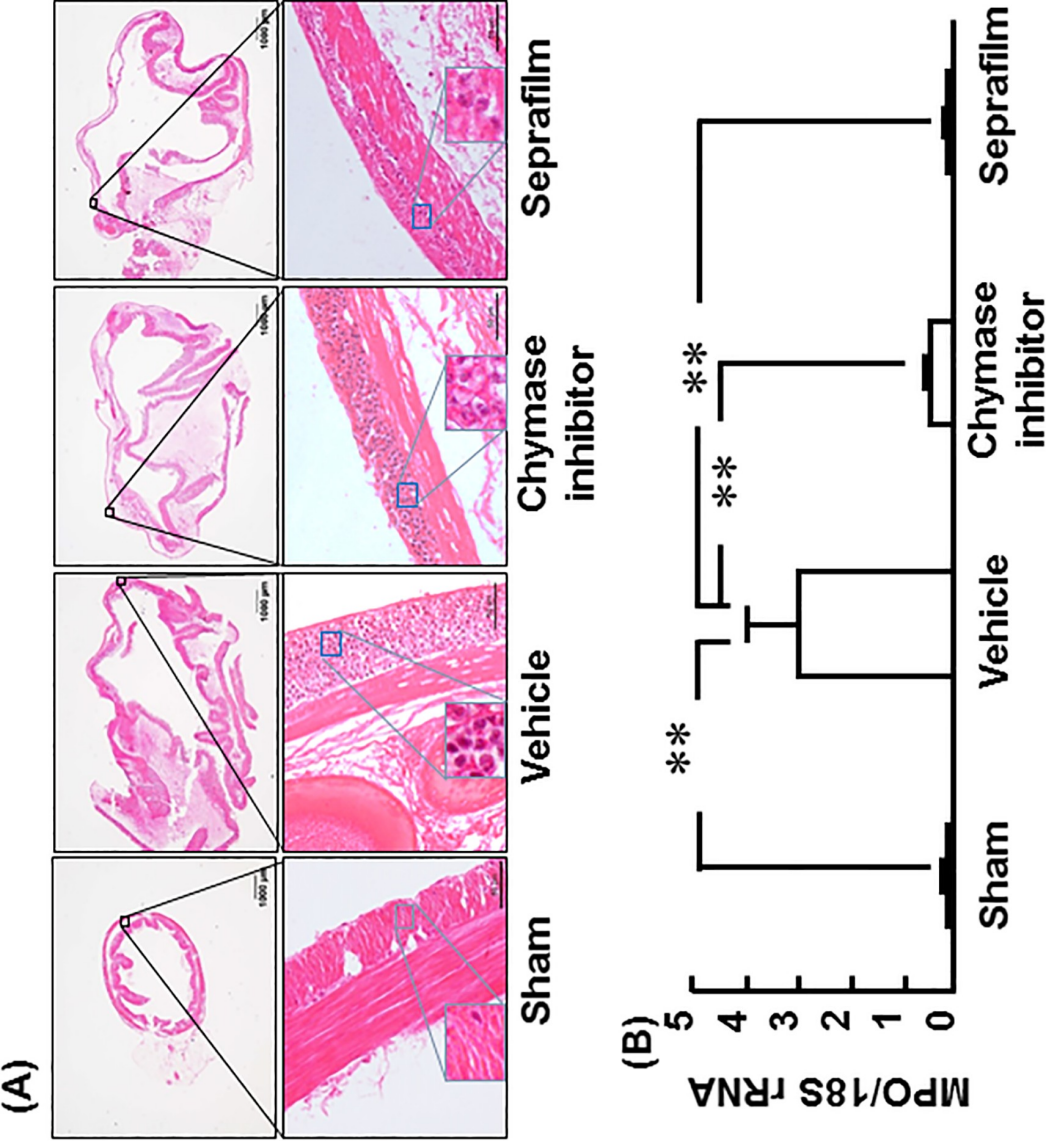
Effects of chymase inhibitor and Seprafilm on MPO mRNA level. Representative images of cecak sections stained with HE-stain in the vehicle-, chymase inhibitor-, and Seprafilm-treated rats 2 weeks after alkali-injury (A). The original magnification is 20X (upper images); scale bars are 1 mm (A). The original magnification is 200X (lower images); scale bars are 0.05 mm (A). Cecal MPO mRNA levels in the vehicle-, chymase inhibitor-, and Seprafilm-treated groups 2 weeks after alkali-injury (B). Values represent means ± SEM (n = 6). **P<0.01 vs. vehicle-treated group.

Representative images of HE-stained cecum in the sham, vehicle, chymase inhibitor and Seprafilm groups 1 day after alkali-induced injury are shown in Fig 5A. Numerous accumulation of inflammatory cells was observed in the vehicle group, but it was clearly reduced in the chymase inhibitor and Seprafilm groups (Fig 5A).

Cecal MPO gene expression was significantly higher in the vehicle group than in the sham group 1 day after alkali-induced injury (Fig 5B). However, cecal MPO gene expression was significantly lower in both the chymase inhibitor and Seprafilm groups than in the vehicle group (Fig 5B).

### Cecal TGF-β protein level

**Fig 6:**
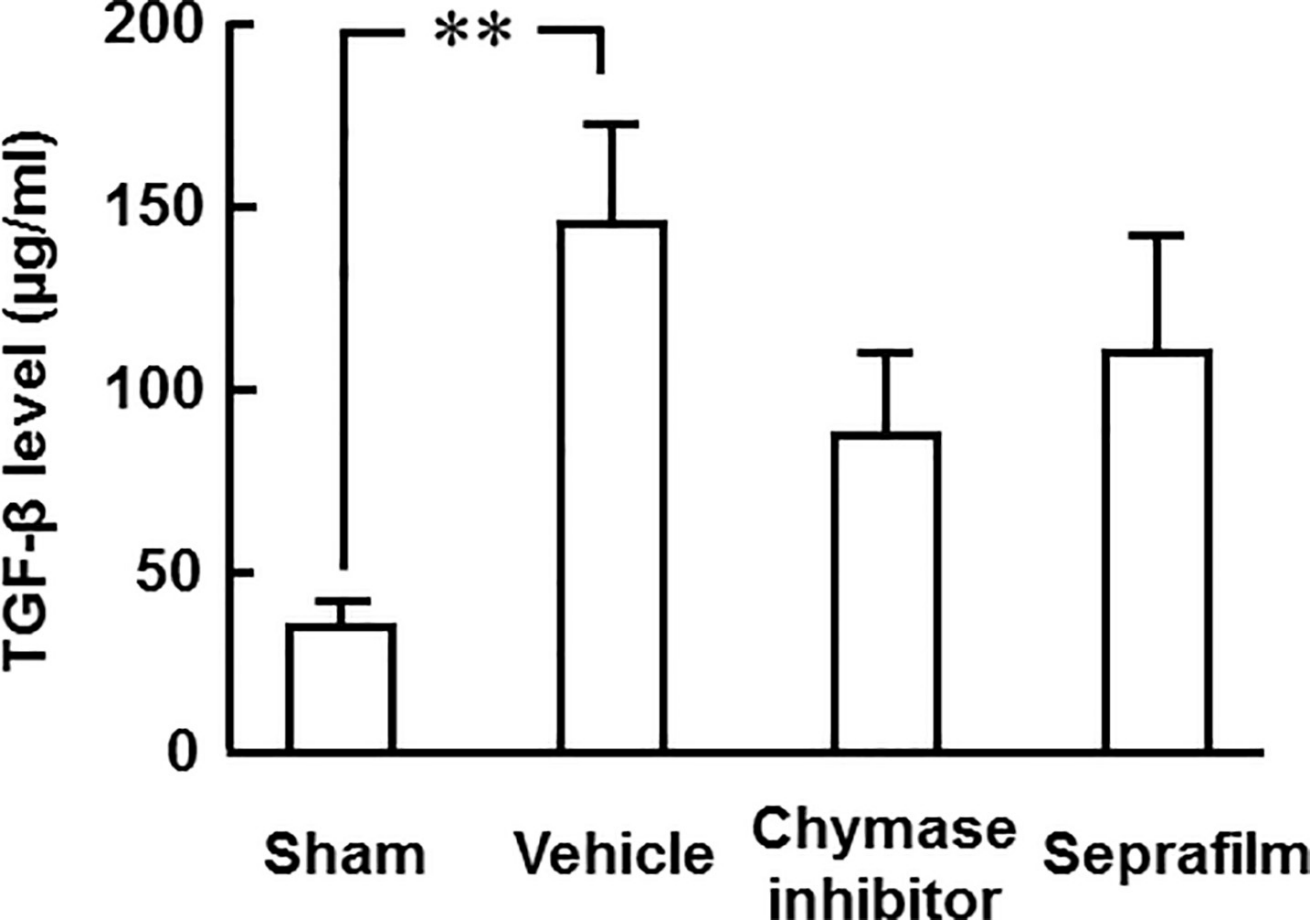
Effects of chymase inhibitor and Seprafilm on TGF-β protein level. Cecal TGF-β protein levels in the vehicle-, chymase inhibitor-, and Seprafilm-treated groups 2 weeks after alkali-injury (B). Values represent means ± SEM (n = 6). **P<0.01 vs. vehicle-treated group.

A significant increase of TGF-β protein levels in the cecum was observed in the vehicle group compared with the sham group 3 days after alkali-induced injury (Fig 6). However, TGF-β protein levels in the cecum tended to be attenuated in the chymase inhibitor and Seprafilm groups compared with the vehicle group (Fig 6).

### Numbers of mast cells and chymase-positive cells in adhesion lesions

**Fig 7:**
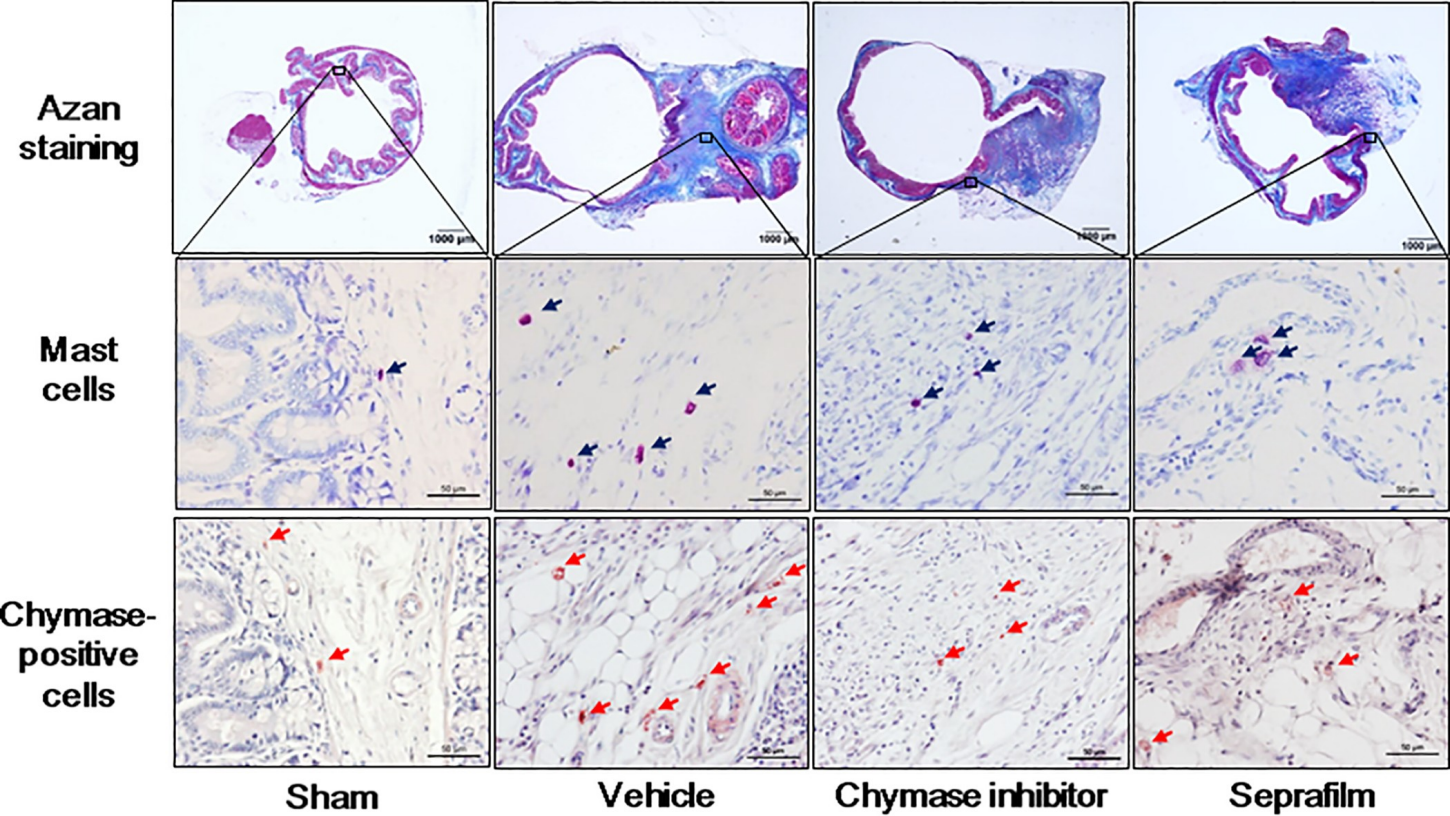
Mast cells and chymase-positive positive cells in cecum. Representative images of cecal sections stained with Azan-stain and toluidine blue (mast cells), and immunostained with anti-chymase (chymase-positive cells) in the sham-, vehicle-, chymase inhibitor-, and Seprafilm-treated rats 2 weeks after alkali-injury. The original magnification is 20X (upper images); scale bars are 1 mm. The original magnification is 200X (middle and lower images); scale bars are 0.05 mm.

Representative images of toluidine blue-stained cells as mast cells and chymase-positive cells in adhesion lesions in the sham, vehicle, chymase inhibitor and Seprafilm groups 2 weeks after alkali-induced injury are shown in are shown in Fig 7.

**Fig 8:**
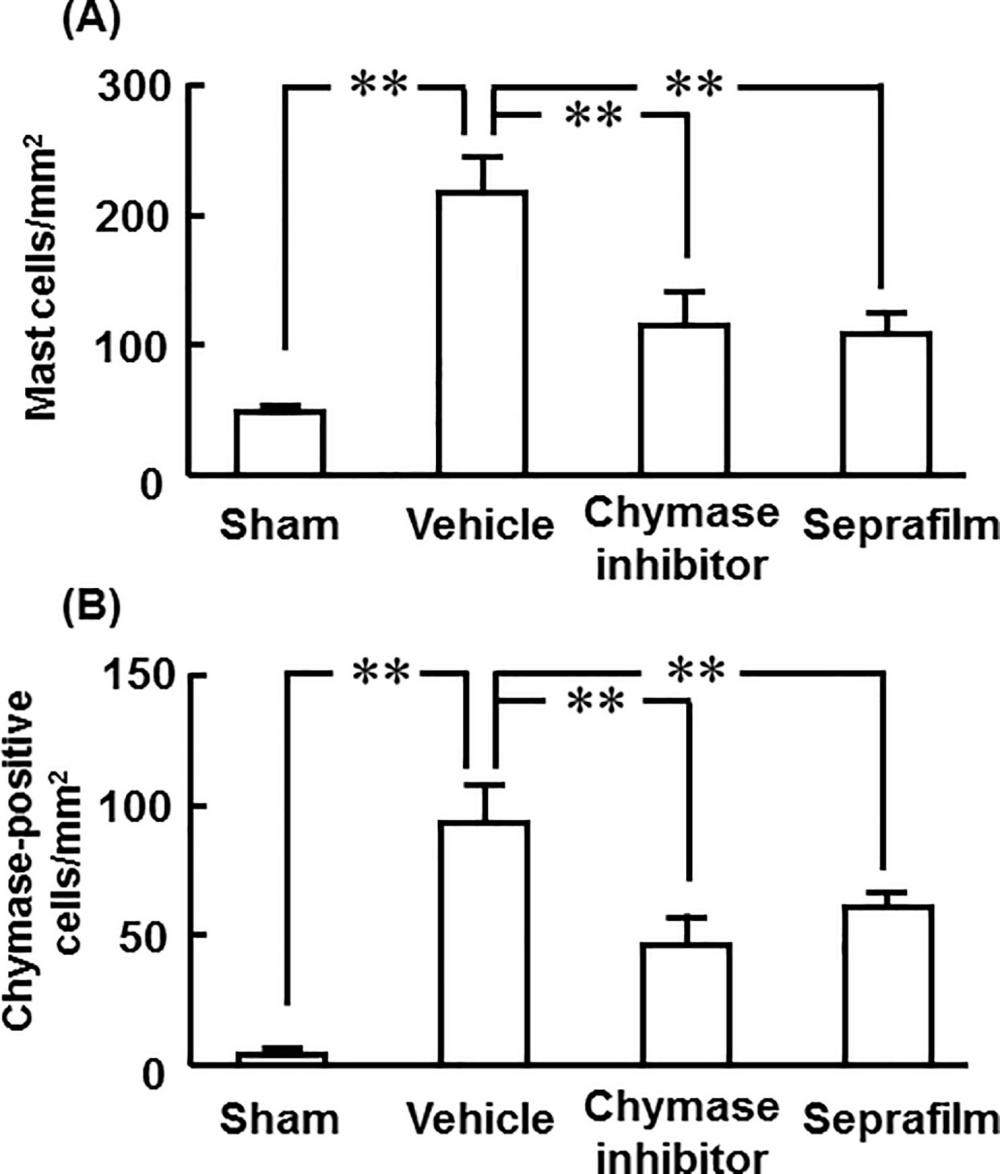
Numbers of mast cells and chymase-positive positive cells in cecum. Numbers of mast cells (A) and chymase-positive cells (B) in cecal sections in the sham-, vehicle-, chymase inhibitor-, and Seprafilm-treated groups 2 weeks after alkali-injury. Values represent means ± SEM (n = 6). **P<0.01 vs. vehicle-treated group.

A significant increase of mast cell numbers was seen in the adhesion lesions in the vehicle group compared with the sham group 2 weeks after the injury, but they were significantly reduced in both the chymase inhibitor and Seprafilm groups (Fig 8A).

The numbers of chymase-positive cells were also significantly higher in the vehicle group than in the sham group, and they were significantly lower in both the chymase inhibitor and Seprafilm groups (Fig 8B).

## Discussion

Previously, we and other groups showed that scarring formation was induced by treatment with alkali in the upper and lower conjunctival sacs, and that severe adhesion formation was also observed in the conjunctiva [22–24]. In reference to this method, a 1 N sodium hydroxide-soaked cotton pad was placed on the cecum for 1 minute. On the other hand, the serous membrane of the cecum was abraded with a swab until punctate hemorrhage occurred as a common adhesion model [15,16]. In the present study, the scores for severity and area 1 and 2 weeks after injury induced by alkali or abrading were assessed. At both time points, both the scores for severity and area were lower in the abrasion-induced model than in the alkali-induced model, and obvious wide variation was observed in the abrasion-induced model compared with the alkali-induced model (Table 1). Moreover, the alkali-induced model may be more objective than the abrasion-induced model, because it is very difficult to ensure the same strength of abrading. In fact, the alkali-induced model showed small adhesion dispersion of severity and area scores. In the alkali-induced model, the scores for severity and area were higher at 2 weeks than at 1 week after alkali-induced injury, and the effects of a chymase inhibitor and Seprafilm were evaluated 2 weeks after alkali-induced injury in the present study.

Severe adhesion formation was observed in the vehicle group, but it was obviously attenuated by treatment with chymase inhibitor and Seprafilm to the same degree. Seprafilm has been used to prevent adhesion formation in clinical and experimental settings [3–5]. However, Seprafilm did not directly affect the function of inflammatory cells such as neutrophils in an in vitro experiment, and it did not affect the systemic inflammatory response [6,25]. Seprafilm has been thought to have no pharmacological effect. Seprafilmhydrates to form a lubricating gel coating within 24 to 48 hours after application to the surface of tissues and is resorbed from the site of application within 1 week [3]. Therefore, the physical barrier of Seprafilm may contribute to the prevention of adhesion formation up to 7 days after application. On the other hand, it has been shown that chymase has several enzyme functions, such as accumulation of inflammatory cells and activation of TGF-β, both of which are associated with the process of adhesion formation [12,26]. In this study, 10 μM Suc-Val-Pro-Phe^p^(OPh)_2_, which prevented adhesion formation in hamsters, was used [13]. The IC_50_ value of Suc-Val-Pro-Phe^p^(OPh)_2_ is 2.8 nM, and its inhibitory mode is irreversible, which means that its inhibitory effect continues for a long time [27,28]. In dogs, the inhibitory effect was maintained for up to 7 days after grafted veins were infiltrated with 10 μM Suc-Val-Pro-Phe^p^(OPh)_2_ only during the operation [29]. The inhibitory effect of the chymase inhibitor may be present for up to 7 days after treatment in vivo.

Significant augmentations of cecal chymase activity were observed 1 and 3 days after alkali-induced injury. However, cecal chymase activity could not be measured because of severe adhesion formation 7 days after the injury. The chymase inhibitor strongly inhibited cecal chymase activity 1 and 3 days after alkali-induced injury. Surprisingly, the strong inhibitory effects of Seprafilm were the same as those of the chymase inhibitor. Seprafilm has no direct pharmacological effect, and it is very difficult to understand the mechanism. However, upregulation of chymase activity may depend on that derived from neighboring normal tissues other than the alkali-injured cecum, because chymase activity was abolished in the cecum just after alkali-induced injury. Unfortunately, mast cells and chymase-positive cells could not be clearly observed on histological analysis 1 day after alkali-induced injury. Not only chymase, but also sulfated proteoglycans are stored in granules of mast cells, and they spread widely after activation of mast cells. The sulfated proteoglycans in secretory granules of mast cells have a metachromatic property of being clearly stained by toluidine blue. Chymase is stored in the granules of mast cells at high concentration and is clearly stained by immunostaining with anti-chymase antibody. However, the activated mast cells may be undetectable by immunostaining with anti-chymase antibody and toluidine blue staining in tissues where there is strong inflammation [30]. Although the decreased chymase activity by the chymase inhibitor is easily understood, the decrease by Seprafilm may depend on the disturbance of recruitment from chymase in neighboring normal tissues by the physical barrier, reducing chymase activity in the injured cecum.

MPO gene expression was evaluated in the very acute phase, 1 day after alkali-induced injury. MPO is thought to reflect accumulation of inflammatory cells such as macrophages and neutrophils, because MPO is expressed in macrophages and neutrophils that are recruited several hours after injury. On histological analysis, accumulation of inflammatory cells was observed 1 day after alkali-induced injury in the vehicle group, but it was significantly attenuated not only by chymase inhibitor, but also by Seprafilm. In several inflammatory experimental models, chymase inhibitor has shown attenuation of inflammatory cells such as macrophages and neutrophils [31,32]. The attenuation of inflammatory cells was significantly reduced not only by chymase inhibitor, but also by Seprafilm. The mechanisms by which a chymase inhibitor and Seprafilm attenuate accumulation of inflammatory cells from neighboring tissues may also be dependent on the pharmacological effect and the physical barrier, respectively.

Three days after alkali-induced injury, filmy adhesions, but not severe adhesions, were seen on the surface of the cecum, and the TGF-β protein level was significantly increased in the cecum in the present study. In vitro, chymase releases the active form of TGF-β from its precursor TGF-β-binding protein, which is not bioactive [12]. TGF-β is known to strongly induce the growth of fibroblasts. In cultured fibroblasts, chymase significantly accelerates fibroblast proliferation via the increase of TGF-β formation [12]. In contrast, the chymase-dependent fibroblast proliferation was completely inhibited by anti-TGF-β neutralizing antibody or 10 μM Suc-Val-Pro-Phe^p^(OPh)_2_ that was used in this study [12]. This finding suggests that chymase promotes fibroblast proliferation via TGF-β activation. TGF-β contributes to the synthesis of extracellular matrix by production of collagen, and its overexpression by peritoneum or increase of TGF-β concentration in peritoneal fluid is associated with an increased incidence of adhesion formation in humans and animals [33–35]. In a rat abdominal adhesion model, significant prevention of adhesion formation was observed with anti-TGF-β neutralizing antibody [36]. The TGF-β protein level in the cecum after alkali injury was attenuated not only by chymase inhibitor, but also by Seprafilm in the present study. The attenuation of cecal TGF-β protein levels by both treatments may contribute to preventing adhesion formation.

At the areas of adhesion, the numbers of both mast cells and chymase-positive cells were significantly increased 2 weeks after alkali-induced injury. Augmentation of mast cells has been observed at adhesion lesions in previous studies [9,13]. In contrast, adhesion formation was prevented after peritoneal injury in mast cell-deficient mice [11]. Chymase has several enzymatic functions, and it is also known to activate stem cell factor (SCF) by proteolytically cleaving the inactive membrane-bound form of SCF to generate the active form of SCF [37]. Chymase-activated SCF stimulates the development and proliferation of mast cells, resulting in increased mast cell numbers [37]. In a previous adhesion model, chymase inhibition resulted in the reduction of mast cell numbers at adhesion lesions [13]. The reason why not only chymase inhibitor but also Seprafilm reduced mast cell numbers at adhesion lesions may be dependent on attenuation of chymase activity in the acute phase after alkali-induced injury.

In a limitation, we evaluated the preventive effects of chymase inhibitor and Seprafilm using the alkali-induced model, but it is difficult to illustrate whether the alkali-induced model or the abrasion-induced model reflects the postoperative abdominal adhesion in clinical setting. There was no severe injury and adhesion other than alkali-injured region in the alkali-induced model, like in the abrasion-induced model. Therefore, other side effect may not be occurred in the alkali-induced model compared with the abrasion-induced model. Moreover, Seprafilm which has been approved for prevention of adhesion formation in clinical setting prevented the adhesion formation in the alkali-induced model as the same findings has been observed in the abrasion-induced model. At least, the alkali-induced model may be useful to evaluate anti-adhesion substances including drugs and sheets.

In conclusion, treatment with either a chymase inhibitor or Seprafilm showed the same degree of adhesion formation in a novel adhesion model that developed severe adhesion formation with high reproducibility. Both the chymase inhibitor and Seprafilm attenuated inflammatory cell numbers and TGF-β levels, in addition to inhibition of chymase activity after abdominal injury, which prevented adhesion formation. A chymase inhibitor solution at laparoscopic surgery may be useful for preventing adhesion formation.

## Supporting information

S1 File This is the ARRIVE guidelines checklist. (PDF)
